# Loss of *Magel2*, a Candidate Gene for Features of Prader-Willi Syndrome, Impairs Reproductive Function in Mice

**DOI:** 10.1371/journal.pone.0004291

**Published:** 2009-01-27

**Authors:** Rebecca E. Mercer, Rachel Wevrick

**Affiliations:** Department of Medical Genetics, University of Alberta, Edmonton, Alberta, Canada; University of Florida, United States of America

## Abstract

**Background:**

*MAGEL2* is one of several genes typically inactivated in the developmental obesity disorder Prader-Willi syndrome (PWS). The physiological consequences of loss of *MAGEL2*, but without the concurrent loss of other PWS genes, are not well understood. Gene-targeted mutation of *Magel2* in mice disrupts circadian rhythm and metabolism causing reduced total activity, reduced weight gain before weaning, and increased adiposity after weaning.

**Principal Findings:**

We now show that loss of *Magel2* in mice causes reduced fertility in both males and females through extended breeding intervals and early reproductive decline and termination. Female *Magel2*-null mice display extended and irregular estrous cycles, while males show decreased testosterone levels, and reduced olfactory preference for female odors.

**Conclusions:**

Our results suggest that loss of *MAGEL2* contributes to the reproductive deficits seen in people with PWS, and further highlights the role of normal circadian rhythm in the maintenance of fertility.

## Introduction

The master pacemaker of circadian rhythm lies in the suprachiasmatic nucleus (SCN) of the hypothalamus. Neurons in the SCN send axonal projections to hypothalamic and non-hypothalamic target regions involved in reproduction, including the medial preoptic area, the gonadotropin-releasing hormone (GnRH) neurons, and the autonomic nervous system. Alterations to circadian rhythm, either through surgical hypothalamic lesions or genetic mutation, have downstream effects on circadian behaviour and hormonal function. In animal models, circadian disruption is often associated with a decrease in reproductive performance. Mutations in key circadian rhythm genes affect female fertility through irregular estrous cycles, pregnancy failure, and reduced survival to weaning in rodents [1]–[3]. Male circadian mutant mice typically have normal reproductive capacity, although *Bmal1* deficiency can cause infertility [4] and *clockΔ19* male mice sire smaller litters [5]. A disruption of the circadian output gene VPAC2R causes an age-related decline in male fertility associated with seminiferous tubular degeneration and associated hypospermia [6].

Altered patterns of sleep and decreased reproductive capacity coincide in several disabling human genetic disorders, including Prader-Willi syndrome (PWS), Smith-Magenis syndrome, and Fragile-X syndrome. PWS is a contiguous gene deletion syndrome generally recognized at birth because of severe hypotonia, hypogonadism, and failure to thrive, followed by developmental delay, hyperphagic obesity, and relative growth hormone deficiency in early childhood [7]. Dysfunction of the autonomic nervous system and altered endocrine function are also characteristic of PWS. Hypogonadotropic hypogonadism typically manifests as hypoplastic external genitalia and delayed gonadal maturation, with delayed menarche and oligomenorrhea in females, and cryptorchidism and hypogonadism in males [8]. While there are two reports of successful pregnancies in women with PWS following hormonal induction, male fertility has never been reported [8]–[10]. Excessive daytime sleepiness and nighttime sleep disruptions frequently occur in PWS, although disruption of the circadian rhythm *per se* has not been described.

People with PWS have congenital loss of function of at least five genes including *MAGEL2*, encoding a member of the MAGE/necdin family of proteins [11], [12]. In mice, *Magel2* is highly expressed in the hypothalamus, and *Magel2* RNA has a circadian profile of expression in the SCN [13], [14]. Mice with a targeted deletion of *Magel2* have altered circadian patterns of food consumption and wheel running activity that point to a deficiency in circadian output from the SCN [13]. We noted reduced weight gain between birth and weaning and increased adiposity in adult *Magel2*-null mice, with reduced food intake and activity levels [15]. We postulated that the hypothalamic defect that we propose causes abnormalities of circadian rhythm and metabolism in *Magel2*-null mice could be accompanied by reduced fertility. We now report that loss of *Magel2* alters reproductive function in both male and female mice.

## Results

*Magel2*-null mice were previously constructed by gene-targeted replacement of the open reading frame of *Magel2* with a lacZ reporter cassette [13], and are maintained on a C57BL/6 background. Mice that inherit the gene-targeted allele from their fathers are “*Magel2*-null” and lack expression of *Magel2* because of genomic imprinting that silences the maternally inherited wild-type allele. Young *Magel2-*null mice are relatively healthy and fertile and display no overt physiological abnormalities, but do display abnormal behavior on formal testing (Mercer *et al.*, submitted). We noted no differences in the anatomy of the external or internal reproductive organs of *Magel2*-null mice at birth, and in particular did not detect any male mice with cryptorchidism.

### Magel2-null females display delayed and lengthened puberty

Female mice were monitored for puberty by inspection for vaginal opening and age at first estrus. *Magel2*-null females display a slight but significant delay of 1.4 days in age at vaginal opening (Table 1, p<0.0004) and an additional delay of 5.3 days in age at first estrus (Table 1, p<0.008) indicating defects in both the initiation and duration of puberty. It has been established that rodents that are underweight at the normal time of puberty can exhibit delayed vaginal opening and delayed onset of estrus [16]. Although *Magel2*-null pups are underweight prior to weaning [15] no significant differences in the weights of the pups were observed during the time of pubertal examination (P28–P40, data not shown).

**Table 1:** Onset of puberty determined by age at vaginal opening and first estrus

	Control (n)	*Magel2-*null (n)	*p-*value
Age at vaginal opening (d)	29.5±0.3 (18)	30.9±0.2 (17)	<0.0004
Age at first estrus (d)	31.3±0.9 (7)	36.6±1.4 (7)	<0.008

Data shown are mean±S.E.M.

### Magel2-null mice show early reproductive decline with infertility by 24 weeks of age

**Figure 1:**
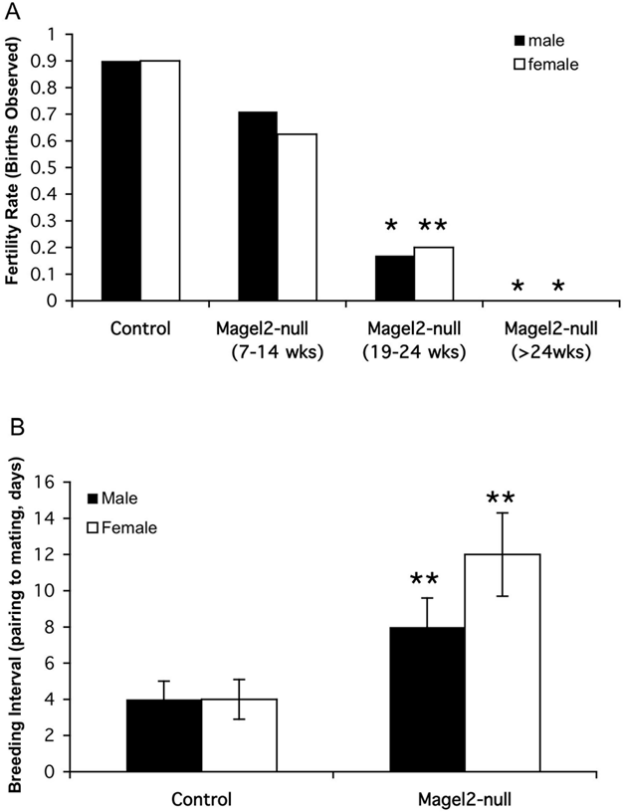
Reduced fertility in *Magel2*-null mice. (A) Fertility rate was measured by pairing mice and monitoring the cages for births, with pairs split when the female mouse was visibly pregnant. The fertility rate is the percentage of pairings that resulted in a litter, for each age group. The control littermate fertility rate includes mice aged 7–35 weeks. The fertility rate for *Magel2*-null mice is split into three age categories as marked. All mice tested were paired with C57BL/6 mice that were 6–10 weeks old. Declining fertility in the *Magel2*-null mice of both sexes is evident by 19 weeks of age, with infertility beyond 24 weeks of age. (B) Breeding interval was determined by subtracting gestational length from the number of days between pairing and birth. Extended breeding intervals are seen in both male and female *Magel2*-null mice. *p<0.001; **p<0.01 for *Magel2*-null versus control, error bars represent SEM.

To test whether loss of *Magel2* affects fertility, we paired either *Magel2-*null mice or their control littermates with 6–10 week old C57BL/6 mice. We then noted whether a litter was born, and the number of days until the litter was born, with pairs split when the female was visibly pregnant. The fertility rate for pairings between control littermate mice and C57BL/6 mice was over 80% (Fig. 1A, control littermates aged 7–35 weeks grouped together). In contrast, pairings between *Magel2*-null and C57BL/6 mice were less successful (Fig. 1A, *Magel2*-null mice split into three age categories). While 71% of C57BL/6 females paired to 7 to 14-week old *Magel2-*null males became pregnant, significantly fewer (17%, p<0.001) of C57BL/6 females paired with 19 to 24-week old *Magel2*-null male mice became pregnant. Furthermore, no pregnancies have been observed in C57BL/6 females paired with *Magel2*-null males older than 24 weeks of age (p<0.001, Figure 1A, n = 12 in each age category). *Magel2*-null females were also less fertile and displayed declining fertility with age. Although 63% of 7 to 14-week old *Magel2-*null females became pregnant when paired with C57BL/6 males, significantly fewer (20%, p<0.01) 19 to 24-week old *Magel2*-null females became pregnant, and no litters have been born to mutant females paired beyond 24 weeks of age, even when housed with fertile C57BL/6 males for over 60 days (p<0.001, Figure 1A, n = 8–10 in each category).

Furthermore, for both male and female *Magel2*-null mice that were eventually successful in breeding, the interval between pairing and birth was significantly extended at all ages. Daily examination of the female mice for the presence of vaginal plugs indicative of mating revealed that as with C57BL/6 mice, most *Magel2*-null females mated with C57BL/6 males and C57BL/6 females mated with *Magel2*-null males had litters within 20 days of the positive plug date, indicating that there was no decreased survival of entire litters. Rather, the mean number of days between pairing and mating was extended to 9 days for pairings involving *Magel2-*null males, compared to the C57BL/6 mean of 4 days that is consistent with the estrous cycle of wild-type female mice (Figure 1B, n = 14 of each genotype). For matings involving *Magel2*-null females, the interval between pairing and mating was further extended to a mean of 12 days (Figure 1B, n = 12 of each genotype).

The mean litter size sired by *Magel2*-null males was similar to the size of litters sired by functionally wild-type *Magel2−/+* male mice carrying a maternally inherited *Magel2-lacZ* knock-in allele, suggesting adequate number of sperm and comparable embryonic viability. Ninety-six percent of pups sired by *Magel2*-null males survive until weaning, a number consistent with the typical 95% weaning rate of control mice in the same environment. In contrast, the average litter size born to *Magel2*-null females was slightly but significantly smaller than controls (*Magel2-*null 6.4 pups versus control 7.8 pups, p<0.05, n = 10–15 litters per genotype), indicating fewer ovulations or increased embryo resorptions. The *Magel2-*null females frequently cannibalized their litters within two days of birth, and we had no litters that survived to weaning born to *Magel2*-null females older than 10 weeks of age (n = 8). Of female *Magel2*-null mice younger than 10 weeks old, we were only able to successfully wean two litters to *Magel2*-null females in our conventional breeding facility, and survival to weaning was 50–60%, much less than the typical weaning rate of 95%. Surviving pups from *Magel2-*null dams were of normal size and weight at weaning, suggesting intact ability of *Magel2*-null female mice to lactate and foster offspring despite low rates of survival of the pups.

### Magel2-null males have reduced testosterone, but normal leutinizing hormone and follicle-stimulating hormone levels

*Magel2* is expressed predominantly in the brain, so effects on reproductive systems presumably originate in the nervous system. Hypothalamic regulation of reproductive function is controlled by GnRH neurons, which stimulate the release of leutinizing hormone (LH) and follicle-stimulating hormone (FSH) from the pituitary. To rule out an intrinsic GnRH neuron deficit, we sectioned perfused brains of 10 week-old female mice, and both 10 and 24 week-old male mice, then performed immunohistochemistry with a GnRH antibody. No difference was observed in neuron number or GnRH content between the *Magel2-*null and control brains at either age, indicating that loss of *Magel2* does not cause loss or displacement of GnRH neurons.

We next measured key reproductive hormones controlled by GnRH secretion in a group of 20–26 week-old male mice. Mean serum testosterone levels were significantly lower in mice lacking *Magel2* (*Magel2-*null 6.1±1.4 ng/ml versus control 20.2±9.9 ng/ml, p<0.03). Subsequent measurement of LH and FSH levels revealed low-normal LH levels (*Magel2*-null 0.12±0.03 ng/ml versus control 0.16±0.04 ng/ml, not significant (n.s)), and normal FSH levels (*Magel2*-null 32.8±2.6 ng/ml versus control 36.8±3.2 ng/ml, n.s).

### Reproductive histology is normal in Magel2-null males but age-related changes are present in ovaries of Magel2-null female mice

**Figure 2:**
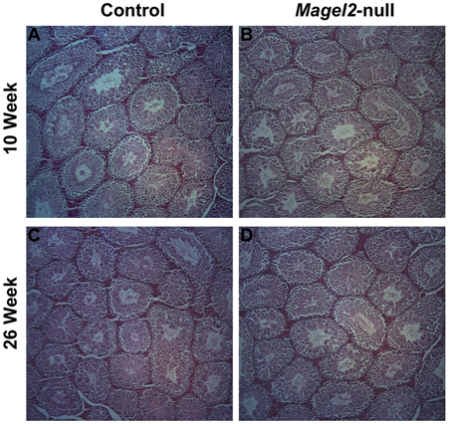
Testicular histology in *Magel2-*null males. (A, C) Hematoxylin and eosin stained paraffin sections of control testes from 10 (A) and 24 week old (C) males. (B, D) Sections from *Magel2*-null males at both 10 (B) and 24 weeks (D) show no difference from control sections.

To determine whether low testosterone levels were associated with histological changes in the testes or impaired spermatogenesis, we compared the testes of control and *Magel2*-null males. At both 10 and 26 weeks of age there was no difference in the weights of the testes between genotypes, and the architecture of the testes and epididymus were histologically normal in *Magel2-*null males (Figure 2). The quantity, morphology, and motility of sperm recovered from the epididymus at both ages were also normal, suggesting that despite reduced testosterone, the male reproductive organs develop and function sufficiently to produce a normal number and quality of gametes.

**Figure 3:**
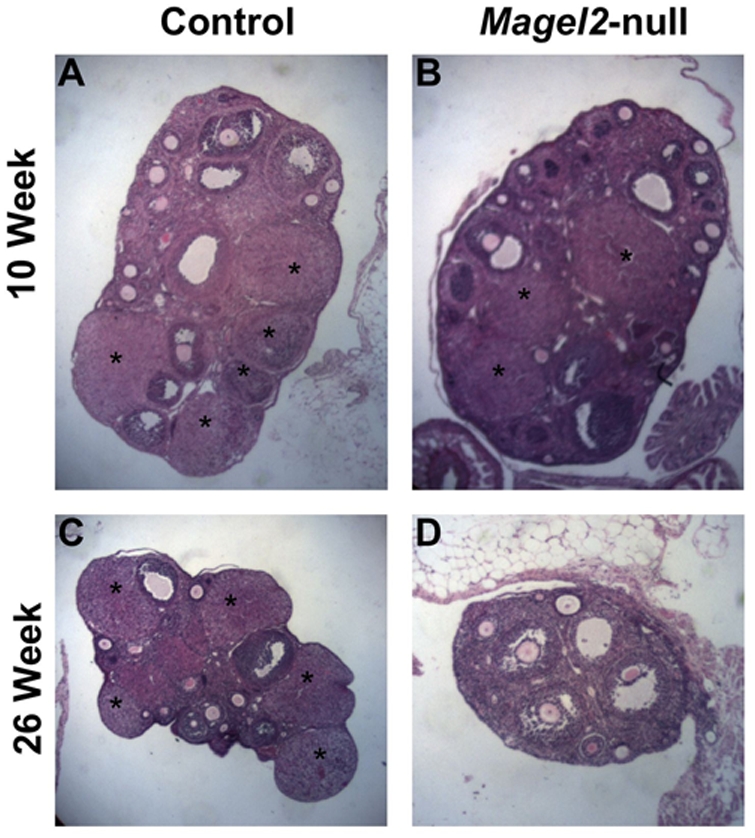
Ovarian histology in *Magel2-*null females. (A, C) Hematoxylin and eosin stained paraffin sections of control ovaries from 10 (A) and 24 week old (C) females. (B, D) Sections from *Magel2*-null females at 10 weeks (B) show no difference from control sections, while sections from 24 week females are notably lacking corpora lutea (indicated by *)(D).

Reduced reproductive rates in the female mice could be explained by a suboptimal uterine environment, early pregnancy failure, impaired folliculogenesis, missed ovulations, or a combination of these events. There was no difference in the gross anatomy or weight of ovaries and uteri collected from *Magel2*-null females at 26 weeks of age. Because female *Magel2*-null mice that were positive for the presence of a vaginal plug had litters at the same frequency as wild-type, and litter size was only slightly reduced, it is unlikely that *Magel2*-null females have any significant uterine changes or early pregnancy losses that could explain their reproductive failure. To examine folliculogenesis, diestrus ovaries from 10- and 24-week *Magel2*-null and control females were collected, and examined for the presence and quantity of developing follicles and corpora lutea. At 10 weeks of age, there was no histological difference between *Magel2*-null and control ovaries, with comparable numbers of Graafian follicles and corpora lutea present (Figure 3 A–B). In contrast, 26-week old *Magel2*-null females showed an absence of corpora lutea in 10/14 ovaries collected, despite having normal numbers of developing and mature follicles, indicating normal folliculogenesis with missed ovulations in most *Magel2-*null females (Figure 3 C–D).

### Magel2-null females have abnormal estrous cycles that worsen with age

**Figure 4:**
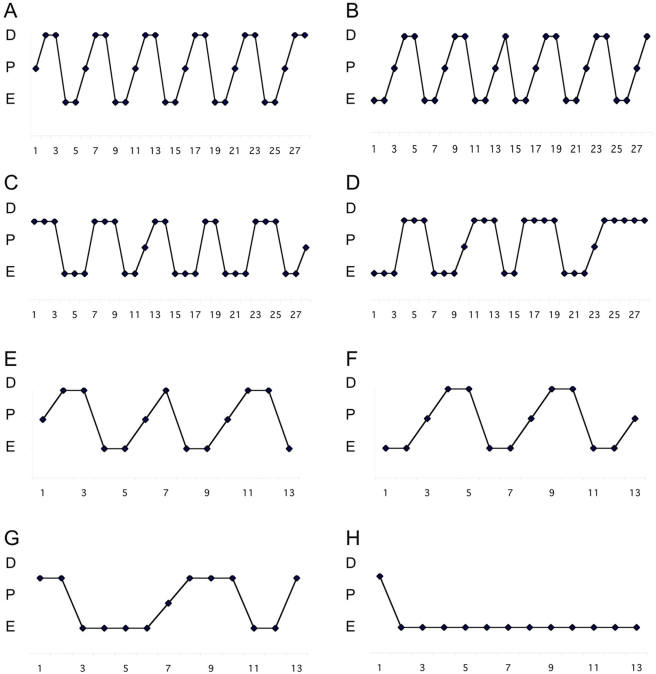
*Magel2*-null females display longer and irregular estrus cycles determined by vaginal cytology. Representative examples of estrous cycling patterns from two individual female mice of each genotype are shown for each of the two ages tested. (A–B) 10 week old control females display regular 4–5 day cycles while 10 week old *Magel*-2 null females spend longer periods in estrus and diestrus, and experience fewer proestrus events (C–D). At 26 weeks of age, control females still display regular cycles (E–F) while *Magel2*-null animals have further deteriorated cycling patterns (G–H). The Y-axis represents estrus stage: E, Estrus; D, Diestrus; P, Proestrus.

A normal mouse estrous cycle is defined as 4–5 days in length, with two days in diestrus, one day in proestrus, and one to two days in estrus [17]. To determine the reason for extended mating intervals in the female mice, we analyzed the regularity of their estrous cycles. Cyclicity was examined by daily sampling of the vaginal epithelium starting at 8 weeks of age for a minimum of 35 days. The stages of estrous can be easily distinguished cytologically, with nucleated smears during proestrus, cornified smears during estrus, and leukocytic smears during diestrus. Representative profiles from individual female mice are shown in Figure 4. The control mice consistently had 4–5 day cycles, with 2 leukocytic smears followed by one nucleated smear and one to two cornified smears (Figure 4 A–B). In contrast, the *Magel2*-null mice had abnormal and extended estrous cycles, with few nucleated smears, and lengthened periods with only cornified smears (Figure 4 C–D). An additional set of 26 week-old *Magel2-*null and control females were also monitored for estrous cyclicity over a 21 day period, and though the control females still displayed regular cycles (Figure 4 E–F), the *Magel2*-null females had even more disruption in their cycling pattern, with only 25% experiencing proestrus (Figure 4 G–H). This type of abnormal cycling is similar to that detected in female mice defective in the *Clock* circadian rhythm protein [1], and is consistent with increased time to mating, reduced fertility with age, and fewer corpora lutea as the ovulatory proestrus period is missed with increased frequency as the female mice age.

### Male Magel2-null mice have reduced olfactory preference for estrous female odor

**Figure 5:**
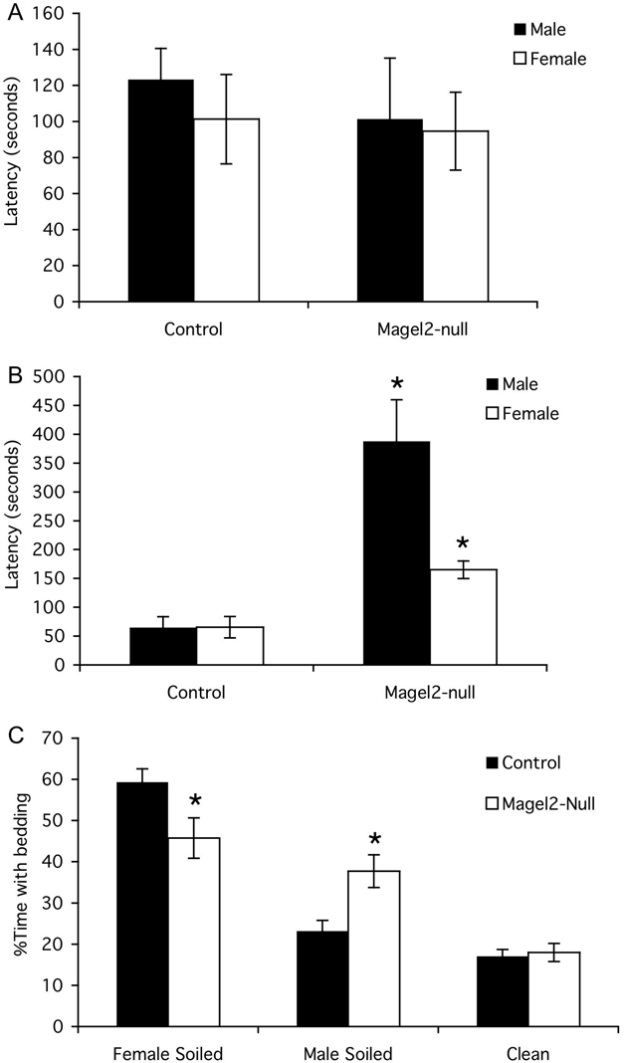
Altered olfaction in *Magel2*-null mice. A) Latency to find buried food tested in 10-week old mice. B) 24 week-old *Magel2*-null mice have increased latency to locate the food following a fast in the buried food olfaction test. C) Three way olfactory preference test. For both genotypes, the percent time spent with clean bedding is less than the percent time with soiled bedding (i.e. the sum of the percent time spent with female-soiled bedding and the percent time spent with male-soiled bedding). However, within the time spent with the two soiled bedding choices, control males prefer female-soiled bedding, while *Magel2*-null males spend an equivalent amount of time with the male-soiled bedding as they do with the female-soiled bedding. * p<0.05 (control vs. *Magel2-*null), error bars represent SEM.

Olfaction is essential for appropriate reproductive behavior in both sexes, and mice with deficient olfaction can also have reproductive deficits [18]. To determine if olfaction is affected in *Magel2-*null mice, we first tested eight male and female mice of each genotype for their ability to find buried food after a 24 h fast. Although 10-week old *Magel2-*null males and females showed no difference in latency to find the buried food compared to control littermates (Figure 5A), at 24 weeks of age both the male and female *Magel2*-null mice took significantly longer to locate the buried food than control mice (Figure 5B). We then measured the length of time that fasted male mice spent investigating a dried vanilla spot painted on the inside of the cage, during a five minute test period. The *Magel2*-null mice spent only 0.75 s investigating the vanilla spot, far less than the mean time of 6.7 s for the control mice (p<0.001, n = 8 mice of each genotype).

To investigate if this apparent olfactory defect affects mating behaviour, we tested olfactory preference in 24 week old male mice by monitoring the time mice spent investigating one of three choices of bedding: 1) bedding soiled by sexually experienced male mice, 2) bedding soiled by female mice in estrus, or 3) unsoiled, clean bedding. We first compared the time spent with soiled bedding (percent of time spent with soiled bedding, sum of choices 1 and 2) to the time spent with clean bedding. Both control and *Magel2*-null males had a clear preference for soiled bedding, spending 82% of their time with soiled bedding and only 17% with clean bedding (Fig. 5C). We then compared the ratio of time spent with bedding soiled by a sexually experienced male versus bedding soiled by an estrus female (choices 1 and 2). Control males showed a clear preference for female-soiled bedding over male-soiled bedding. In contrast, the *Magel2*-null males showed no preference for either male-soiled or female-soiled bedding, spending a similar percentage of their investigation time with either of these two choices (Figure 5C). A similar olfactory preference test was administered to female *Magel2*-null mice at 24 weeks of age, revealing no difference in olfactory preference between control and *Magel2*-null females (data not shown).

We conclude that loss of *Magel2* causes reduced reproductive fitness in both male and female mice, with early-onset decline in fertility, irregular estrus cycles in females, and altered olfactory preference in males.

## Discussion

The aim of these studies was to observe reproductive changes in mice following the loss of the PWS candidate gene *Magel2*, which had previously been shown to cause hypothalamic dysfunction in the areas of circadian function [13] and metabolism [15]. We now show that loss of *Magel2* affects both male and female reproduction, causing reduced fertility and early infertility in both males and females, delayed puberty and irregular estrus cycles in females, and low testosterone, and impaired olfaction and olfactory preference in males.

Young *Magel2-*null mice of both sexes are able to breed, producing relatively normal litters. We previously reported some reduced fertility in male mice with only preliminary investigation in female mice [13]. Normal C57BL/6 mice show an age-related decline in fertility beginning at about 6 months of age, with acyclicity in females beginning around 12 months of age [17]. This decline is typically associated with histological changes including vacuolization of seminiferous tubules and increased Leydig cell number in males [19] and overall loss of follicles and a reduction in developing follicles in females [20]. Because the early loss of fertility seen in our mice was not accompanied by these histological changes in the gonads, it does not seem that loss of reproductive ability is linked to accelerating aging in the testes or ovaries in mice lacking *Magel2*.

*Magel2*-null females have a delay in the onset of puberty, contrasting with the *ClockΔ19* females that display normal timing of both vaginal opening and vaginal estrus [1]. The onset of puberty in other circadian mutant mice has not been described, though an isolated report suggests a slight delay in puberty in *Bmal1* knockout mice [21]. We have previously reported an early failure to thrive in *Magel2*-null neonates followed by adult-onset weight gain and increased adiposity [15], but this weight difference is no longer apparent at the normal timing of puberty. Intriguingly, it has been shown that protein restriction during neonatal development can impair adult fertility by delaying puberty and accelerating reproductive decline in female rats [22], so it is possible that the early failure-to-thrive seen in *Magel2*-null pups contributes to both their delay in puberty and their later reproductive difficulties, but is unlikely to be the sole cause of infertility.

Abnormal estrous cycles in rodents can result from perturbations at any level of the hypothalamic-pituitary-gonadal axis. Because *Magel2*-null females show no anatomical differences of external or internal reproductive organs, and their ovaries have grossly normal folliculogenesis with mature Graafian follicles, ovarian steroidogenesis is not likely perturbed and is therefore not a likely culprit for abnormal estrous cycling in these mice. Rising levels of estrogens produced by developing follicles in the ovary feed back to the hypothalamus, amplifying GnRH pulses, and to the pituitary, sensitizing it to increasing GnRH. The combination of these hypothalamic and pituitary effects result in a precisely timed surge of LH on the afternoon of proestrus, which leads to ovulation some 10–12 h later [17]. Cytological examination of the vaginal epithelium revealed few proestrus events in *Magel2*-null females, coupled with reduced or no corpora lutea in the ovary suggestive of missed ovulation. Normal folliculogenesis in the female *Magel2*-null mice, and normal pituitary hormone levels (LH, FSH) in *Magel2*-null males, suggests a defect in the hypothalamic response to rising estrogen levels, perturbing the precision of the reproductive system. Poor responsiveness could occur despite normal numbers and placement of GnRH neurons. Abnormal estrous profiles and differential ovulation rates have consistently been shown in the *ClockΔ19* mice [1], [2], [5], which do not show a LH surge following proestrus [1]. Like the *Magel2*-null females we describe here, the *ClockΔ19* mice also have an approximate one pup decrease in litter size, which has been attributed to a reduction in the number of ova per ovulation [2], but how these ovulations are occurring in the absence of an LH surge remains unexplained.

By the time male *Magel2*-null mice become functionally infertile around the age of 24 weeks, they have significantly lower testosterone levels than controls. Testosterone is produced by Leydig cells in the testes following stimulation by LH pulses from the pituitary gland, which are regulated by rhythmic pulses of GnRH controlled by the SCN. Low testosterone and infertility have been reported in mice lacking *Bmal1*, a core circadian gene also expressed in Leydig cells. These mice have a threefold increase in serum LH, which indicates a defect in the ability of Leydig cells to produce testosterone [4]. *Magel2* is not expressed in adult mouse testes [12], and we did not detect *Magel2-*LacZ reporter gene expression in Leydig cells in adult *Magel2*-null mice (data not shown). Serum LH levels were not significantly different in the *Magel2-*null males compared to controls, which indicates impaired hypothalamic or pituitary response to the reduced testosterone seen in these mice. Because the structure of the GnRH system appears intact in *Magel2*-null mice, and no testicular abnormalities were detected, abnormal regulation between the central and peripheral components of the hypothalamic-pituitary-gonadal axis is a possible explanation for reduced testosterone levels in the *Magel2*-null mice.

Sexual motivation and successful reproduction in rodents is highly dependent on olfactory ability, with anosmic mice failing to breed [18]. Normal male rodents also show a clear preference for estrus female odors compared to male odors, and rats that do not display an olfactory preference for females generally do not copulate [23]. *Magel2*-null mice of both sexes develop an olfactory defect between 10 and 24 weeks of age as evidenced by increased time to find food following a fast. Because the null mice also showed reduced foraging and digging during these tests, and because they are generally hypoactive [13], [15] the increased time cannot specifically be attributed to an olfactory defect, and may partially result from decreased food-motivation. Consistent with this idea, during the olfactory preference tests, *Magel2-*null mice clearly preferred soiled bedding over clean bedding, indicating functional olfactory detection. However, while control male mice prefer estrus female odor, *Magel2*-null mice did not discriminate between bedding soiled by males or soiled by females, indicating a defect in the detection or response to pheromone cues. Pheromone detection in the mouse has been shown to be a result of a direct neural connection between the main olfactory epithelium and the hypothalamic GnRH neuron system [18], [24], warranting additional examination of the olfactory system in *Magel2*-null mice. Low sexual motivation resulting from reduced testosterone and an inability to respond to female odor cues is the most likely explanation for impaired reproductive function in *Magel2*-null males.

The reduced fertility of *Magel2*-null mice is consistent with that seen in other circadian mutant mice, but subtler than the infertility and incomplete sexual maturation seen in PWS. Delayed and/or partial puberty is often observed in girls with PWS, with late-onset or absent menarche, and amenorrhea or oligomenorrhea in most cases [9], [10]. Occasional cases of premature pubarche and true precocious puberty have been described in a proportion of PWS individuals, usually occurring secondary to obesity [10]. Males with Prader-Willi syndrome typically have hypogonadism, and display Sertoli-only histology in their seminiferous tubules [8], [25], [26]. A recent study of hormone levels in people with PWS aged 16 years and older demonstrated testosterone levels below the normative range in 19 of 23 males, and LH levels below the normative range in 5 of 24 males, while FSH levels were variably low, normal, or high in the same cohort [27]. Because several genes are lost in PWS, combined loss of *Magel2* and other Prader-Willi candidate genes may have an additive deleterious effect. For example, mice lacking a different PWS gene encoding a second MAGE protein, necdin, show a significant reduction in GnRH neuron number in the hypothalamus [28], [29]. Loss of GnRH neuron number, consequent to loss of necdin, coupled with loss of proper feedback circuitry from loss of MAGEL2, would be predicted to have a more serious effect on reproductive function in individuals with PWS with congenital absence of both genes. Further, failure to thrive associated with loss of the snoRNA HBII-85 could contribute to later infertility in PWS, although fertility was reportedly normal in two independent strains of mice deficient for the murine ortholog MBII-85/Snord116 [30], [31].

Further studies on reproductive and other hypothalamic functions in *Magel2*-null mice under conditions of worsened (e.g. through exposure to constant darkness) or improved (e.g. by pharmacological reinforcement of the circadian period or more stringent photoperiods) circadian function may provide compelling evidence for additional examination and regulation of circadian rhythm in people with PWS. Our results further highlight the role of *Magel2* in normal hypothalamic function, and add additional support for the role of circadian rhythm in reproduction.

## Materials and Methods

### Mouse Breeding and Handling

Animal procedures were approved by the University of Alberta Animal Policy and Welfare Committee. The *Magel2* mouse colony was maintained on a C57Bl/6 background by breeding Magel2−/+ female mice carrying a maternally inherited *Magel2-lacZ* knock-in allele with C57Bl/6 male mice to generate heterozygous, functionally wild-type offspring. Because of imprinting that silences the maternally inherited allele, *Magel2*-null mice retain expression only from the paternally inherited *lacZ* knock-in allele [13]. C57Bl/6 female mice were bred with Magel2−/+ male mice carrying a maternally inherited *Magel2-lacZ* knock-in allele. This cross generated Magel2+/− mice carrying a paternally inherited *lacZ* knock-in allele (*Magel2-*null, no expression of *Magel2*) and Magel2 +/+ (control littermate) offspring. Mice were genotyped from tissue samples or ear notch biopsies as described [32]. Mice were weaned between three and four weeks of age unless otherwise stated and then housed 3–4 per cage with food (PicoLab Mouse Diet 20, LabDiet) and water *ad libitum,* and maintained under 12∶12 light dark conditions.

### Puberty and Estrous Cycle Examinations

Female mice used for timing of puberty were weaned at 21 days of age, and inspected daily for the occurrence of vaginal opening. Beginning on the day of vaginal opening, vaginal smears were obtained from the mice using a fine tipped swab as described [17]. Smears were briefly stained in Modified Wright Stain (WS16, Sigma-Aldrich, St. Louis, MO, USA) and examined under a light microscope, with the observer blind to the genotype of the mouse. Smears were characterized as diestrus if mostly polymorphonuclear leukocytes were present, proestrus when mostly small nucleated and some cornified cells were present, and estrus when the smear was completely cornified. Age at first estrus was recorded as the day when the first fully cornified smear occurred. Beginning at 9 or 26 weeks of age, daily vaginal smears were taken to monitor estrous cycles, for a minimum of 21 days. Representative data from individual mice are shown in Fig. 4. For fertility measurements, mice were paired at specific ages and monitored for pregnancy. Males were removed when the female was visibly pregnant, and the cage monitored for the timing of birth of the litter.

### Gonadal Histology

Testes and ovaries were dissected from *Magel2*-null and control mice at 10 and 24–26 weeks of age. Tissues were fixed in 10% neutral buffered formalin (Sigma-Aldrich HT50-1-1, St. Louis, MO, USA), processed into paraffin, sectioned at 5–8 µm, and stained with hematoxylin and eosin for histological analysis. Sections were prepared by the Histology Core Facility of the Alberta Diabetes Institute, Edmonton, Alberta.

### Immunohistochemistry of GnRH in the Brain

For immunohistochemistry, brains from 10- or 26-week old control and *Magel2*-null mice were perfused and fixed in 4% paraformaldehyde, and cut coronally into 150 µm sections using a vibratome. Sections were left at 4°C in 25% sucrose in PBS for 24 h to dehydrate. Sections were washed 3 times with PBS, and then incubated with a GnRH antibody (PA1-121, Affinity Bioreagents, Golden, CO, USA, diluted 1∶1000) in 2% normal goat serum with 0.3% TritonX-100 at 4°C with gentle shaking for 48 h. Sections were washed in PBS, and then incubated with a goat anti-rabbit secondary antibody labeled with Alexa Fluor 594 (Invitrogen, Carlsbad, CA, USA) at 1∶1000 dilution in 2% normal goat serum and 0.3% TritonX-100 for 2 h at room temperature in the dark. Sections were then washed thoroughly with PBS, mounted on slides using ProLong Gold (Invitrogen, Carlsbad, CA, USA), and analyzed by confocal microscopy.

### Hormonal Assays

Blood for analysis of reproductive hormones was obtained by cardiac puncture. Serum was purified by centrifugation, and stored at −20°C until radioimmunoassay was performed. Testosterone, LH, and FSH levels were analyzed by the University of Virginia Centre for Research in Reproduction Ligand Assay and Analysis Core (supported by the Eunice Kennedy Shriver NICHD/NIH Grant U54-HD28934).

### Olfaction Tests

On the first day of the buried food test, a 2cm^3^ piece of sharp cheddar cheese was placed in each mouse's home cage. The following day, mice were subjected to a 24 h fast beginning just before lights out. After this 24 h period, the mice were habituated to clean 29×19×13 cm filter top cages with approximately 5 cm deep bedding for a minimum of 20 min. The mouse was then briefly removed and an approximately 2cm^3^ piece of sharp cheddar cheese was buried under the bedding with random placement along a cage side, avoiding the corners. The mouse was reintroduced to the cage and latency to find the cheese was recorded. Each mouse was tested three times with a minimum 48 h period separating test periods.

A three choice olfactory preference test was administered to assess pheromone odor preference. Each mouse was tested in a clean 48×26×21 cm plexiglass cage containing 3 small (7×7×3 cm) containers filled with either clean bedding (unused autoclaved wood shavings), male-soiled bedding (collected and pooled from cages containing singly housed sexually experienced C57BL/6 males), or female-soiled bedding (collected and pooled from cages housing 3–4 female mice with at least 2 in the active state of estrous as assessed by vaginal appearance [33]). Bedding was used on the same day it was collected. These containers were placed randomly on either side and in the centre of the cage. Mice were habituated to the test arena for a minimum of 10 min. prior to introduction of the olfactory stimuli. The time spent by each mouse investigating each type of bedding was recorded over a 10 min. time period. For each mouse, the time spent with each of the three containers was divided by the total time spent investigating the containers to arrive at a percent time with each choice of bedding.

### Statistical Analysis

Fertility rates were analyzed by Fisher's exact test. Testosterone, LH, and FSH levels were compared using a two-tailed Mann-Whitney U test. Otherwise, statistical analyses of differences between genotypes were performed using the two-way ANOVA function of the GraphPad Prism 4 software package, or Student's paired t-tests. Differences with p<0.05 after correction for multiple t-testing were considered significant.
